# RNA-sequencing analysis reveals novel genes involved in the different peel color formation in eggplant

**DOI:** 10.1093/hr/uhad181

**Published:** 2023-09-04

**Authors:** Jing Li, Senlin Jiang, Guobin Yang, Yanwei Xu, Lujun Li, Fengjuan Yang

**Affiliations:** College of Horticulture Science and Engineering, Shandong Agricultural University/State Key Laboratory of Crop Biology, Tai’an, Shandong 271018, China; Key Laboratory of Biology and Genetic Improvement of Horticultural Crops (Huanghuai Region), Ministry of Agriculture and Rural Affairs, Tai’an, Shandong 271018, China; Shandong Collaborative Innovation Center for Fruit and Vegetable Production with High Quality and Efficiency, Shandong Agricultural University, Tai’an, Shandong 271018, China; College of Horticulture Science and Engineering, Shandong Agricultural University/State Key Laboratory of Crop Biology, Tai’an, Shandong 271018, China; College of Horticulture Science and Engineering, Shandong Agricultural University/State Key Laboratory of Crop Biology, Tai’an, Shandong 271018, China; College of Horticulture Science and Engineering, Shandong Agricultural University/State Key Laboratory of Crop Biology, Tai’an, Shandong 271018, China; College of Horticulture Science and Engineering, Shandong Agricultural University/State Key Laboratory of Crop Biology, Tai’an, Shandong 271018, China; College of Horticulture Science and Engineering, Shandong Agricultural University/State Key Laboratory of Crop Biology, Tai’an, Shandong 271018, China; Key Laboratory of Biology and Genetic Improvement of Horticultural Crops (Huanghuai Region), Ministry of Agriculture and Rural Affairs, Tai’an, Shandong 271018, China; Shandong Collaborative Innovation Center for Fruit and Vegetable Production with High Quality and Efficiency, Shandong Agricultural University, Tai’an, Shandong 271018, China

## Abstract

Eggplant (*Solanum melongena* L.) is a highly nutritious vegetable. Here, the molecular mechanism of color formation in eggplants was determined using six eggplant cultivars with different peel colors and two *SmMYB113*-overexpressing transgenic eggplants with a purple peel and pulp. Significant differentially expressed genes (DEGs) were identified by RNA-sequencing analysis using the following criteria: log_2_^(sample1/sample2)^ ≥ 0.75 and q-value ≤ 0.05. Two analytical strategies were used to identify genes related to the different peel color according to the peel color, flavonoids content, delphinidins/flavonoids ratio, and the content of anthocyanins. Finally, 27 novel genes were identified to be related to the color difference among eggplant peels and 32 novel genes were identified to be related to anthocyanin biosynthesis and regulated by SmMYB113. Venn analysis revealed that *SmCytb5*, *SmGST*, *SmMATE*, *SmASAT3*, and *SmF3′5’M* were shared among both sets of novel genes. Transient expression assay in tobacco suggested that these five genes were not sufficient for inducing anthocyanin biosynthesis alone, but they play important roles in anthocyanin accumulation in eggplant peels. Yeast one-hybrid, electrophoretic mobility shift assay and dual-luciferase assays indicated that the expression of the five genes could be directly activated by SmMYB113 protein. Finally, a regulatory model for the mechanism of color formation in eggplant was proposed. Overall, the results of this study provide useful information that enhances our understanding of the molecular mechanism underlying the different color formation in eggplant.

## Introduction

Anthocyanins are a class of water-soluble flavonoids that are widespread in fruits and vegetables. Anthocyanins are responsible for the colors of fruits and vegetables, including red, purple, and blue. Red to purplish blue-colored and edible leafy vegetables, tubers, roots, and grains provide high nutritional and health benefits [1].

The anthocyanin biosynthesis pathway in plants has been well studied; anthocyanin biosynthesis is catalyzed by *CHI*, *CHS*, *F3H*, *F3’H*, *F3'5'H*, *DFR*, and *ANS*/*LDOX* orderly, named as anthocyanin biosynthesis structural genes, and the anthocyanidins are converted into stable anthocyanins via sugars and acyl acids. In addition, the regulatory effects of the MBW complex on anthocyanin biosynthesis structural genes have been well studied; the main regulatory proteins include MYB, bHLH, and WD40 transcription factors [2]. Within the MBW complex, the MYB transcription factor directly activates or represses the expression of anthocyanin biosynthesis structural genes; the bHLH transcription factor can interact with the MYB transcription factor to enhance the activation effect, and the role of the WD40 transcription factor is to stabilize the structure of the MBW complex.

Recently, many studies have reported that the color differences of fruits, flowers, vegetables, and roots in the same plant species mainly stem from the content and structure of anthocyanins [3–7]. For example, the purple grains of the wheat cultivar XY22p contain more pelargonidin and cyanidin and less delphinidin than the other wheat grains cultivars; the blue grains of the wheat cultivar XY22b contain more delphinidin and less pelargonidin and cyanidin [5]. In *Asparagus officinalis* L., malvidin, malvidin 3,5-diglucoside, pelargonidin, pelargonidin 3-*O*-beta-D-glucoside, and pelargonidin 3-*O*-malonyl-malonylhexoside were only detected in three purple cultivars but not in the green cultivar [6]. The delphinidin 3-*O*-glucoside content and total anthocyanin content are higher in black-purple eggplant peel, while the delphinidin 3-*O*-rutinoside content is highest in reddish-purple eggplant peel [7]. In *Hibiscus syriacus* L. flowers, the total anthocyanins content identified by qualitative and quantitative mass spectrometry analyses was similar between HSR*_red_* and HSPU*_purple_* with values of 1477.489 μg·g^−1^ and 1406.485 μg·g^−1^, but the anthocyanidin derivatives detected near the center of HSR*_red_* were cyanidin and peonidin; the derivatives of malvidin, delphinidin, and petunidin are mainly found in the center of HSPU*_purple_* [3].

To investigate the molecular mechanism underlying differences in color formation within plant species, transcriptomic analyses have been widely used because of their ability to provide insights into the mechanisms underlying complex biological phenomena. For example, the up-regulation of *F3’H*/*F3’5’H* and *TaMYC1*/*TaMYC4* is important for the formation of purple/blue grains [5]. Various anthocyanin biosynthesis genes (*MeF3H*, *MeF3’5’H*, *MeDFR1*, *MeANS*, *MeANR*, *MeMYB5*, and *MeMYB42*) are co-expressed during anthocyanin biosynthesis in yellow-rooted cassava [4]. Similarly, the expression levels of anthocyanin biosynthesis genes are significantly higher in red- and purple-colored radish cultivars than in white and black radish accessions [8]. Our previous studies have reported that anthocyanin biosynthesis is regulated by light in eggplant; aside from several anthocyanin biosynthesis genes previously identified, three new MYB members (*SmMYB35*, *SmMYB44*, and a *SmMYB86* isoform) might be involved in the anthocyanin biosynthesis pathway according to a transcriptomic analysis and transient expression assay [9, 10]. The biological functions and molecular mechanisms by which *SmMYB35* and *SmMYB86* regulate anthocyanin biosynthesis have been investigated in eggplant [11, 12]. Homolog of *SmMYB44*, *IbMYB44* from purple-fleshed sweet potato has been reported to decrease anthocyanin accumulation by competitively inhibiting the formation of the MYB340-bHLH2-NAC56 complex [13].

Eggplant is an economically important vegetable with different peel colors, which could be divided into reddish-purple, black-purple, lavender, orange, white, and green. Purple eggplant fruits are a rich source of anthocyanins, and the ratio of delphinidins/flavonoids is closely associated with the purple peel color [7]. SmMYB113 plays an important role in regulating anthocyanin biosynthesis in eggplant. The anthocyanins in eggplant can be activated by *SmMYB113* overexpression, and delphinidins are the most dominant anthocyanins [7]. The expression of *SmCHS* and *SmDFR* is reported to be regulated by SmMYB113 [14, 15]; however, the two enzymes encoded by *SmCHS* and *SmDFR* are not directly responsible for mediating the synthesis of cyanidins and delphinidins in this pathway. Therefore, the molecular mechanism underlying the different color formation in eggplant remains unclear.

In this study, RNA-seq analysis was carried out to identify differentially expressed genes (DEGs) related to differences in color formation in eggplant peels and the delphinidin biosynthesis pathway regulated by SmMYB113. The results of our study enhance our understanding of the transcriptional regulation of anthocyanin accumulation and will aid the molecular breeding of eggplant.

## Results

### The expression patterns of anthocyanin biosynthesis genes in eggplants of different colors and at different developmental stages

**Figure 1:**
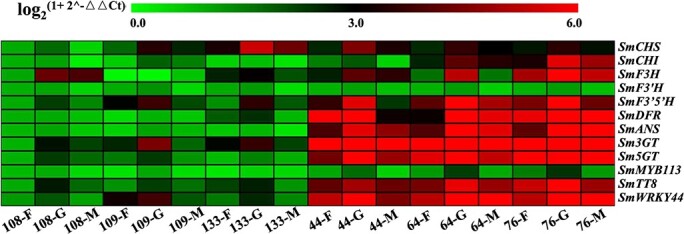
The expression levels of anthocyanin biosynthesis-related genes in the peels of six eggplant cultivars at the fruit setting (F), rapid growth (G), and commodity maturity (M) stages.

To explore the molecular mechanism of the different color formation among eggplant peels, six eggplant cultivars with different peel colors (No. 44, No. 64, No. 76, No. 108, No. 109, and No. 133) were sampled, and these were the same materials sampled in our previous study [7] (Supplementary Data Fig. S1A). The expression patterns of anthocyanin biosynthesis genes in the peels of the six eggplant cultivars were measured during the fruit setting (F), rapid growth (G), and commodity maturity (M) stages, including anthocyanin biosynthesis structural genes and anthocyanin biosynthesis regulatory genes. As shown in Fig. 1, the expression levels of all the anthocyanin biosynthesis genes were nearly all higher in No. 44, No. 64, and No. 76 (with purple peels) than in No. 108, No. 109, and No. 133 (without purple peels), and the expression of all these genes peaked at the G stage of fruits from the six eggplant cultivars excepting *SmF3H* in cultivar No. 109, indicating that the anthocyanin biosynthesis pathway was the most active at the G stage. Therefore, RNA-seq analysis was conducted on eggplant peels at the G stage to investigate the underling molecular mechanism of the different color formation.

### RNA-seq data, RNA quality, and DEGs analysis

After data from each sample were filtered and subjected to quality control procedures, reliable and high-quality sequence data among the three biological replicates were obtained. An overview of the quality of the reads of the different samples is provided in Supplementary Data Table S2. In the libraries, the clean reads percentage was greater than 99%, and the average Q20 and Q30 values were greater than 97.48% and 93.28%, respectively. The genome alignment rates of the 18 libraries ranged from 74.42% to 85.61%. In addition, 68.38% to 73.62% of the high-quality reads per library could be uniquely mapped to published gene data [16]. A total of 30 860 genes were obtained from the 34 samples. DEGs were identified based on the absolute fold change value of log_2_^(sample1/sample2)^ ≥ 0.75 and q-value ≤ 0.05.

In addition, the correlations in the expression patterns of the anthocyanin biosynthesis genes inferred by the qRT-PCR and RNA-seq analyses were high (r = 0.82–0.99) (Supplementary Data Fig. S2). These results indicate that the RNA-seq data were reliable.

### Identification of genes involved in flavonoids and anthocyanins biosynthesis in eggplant

**Figure 2:**
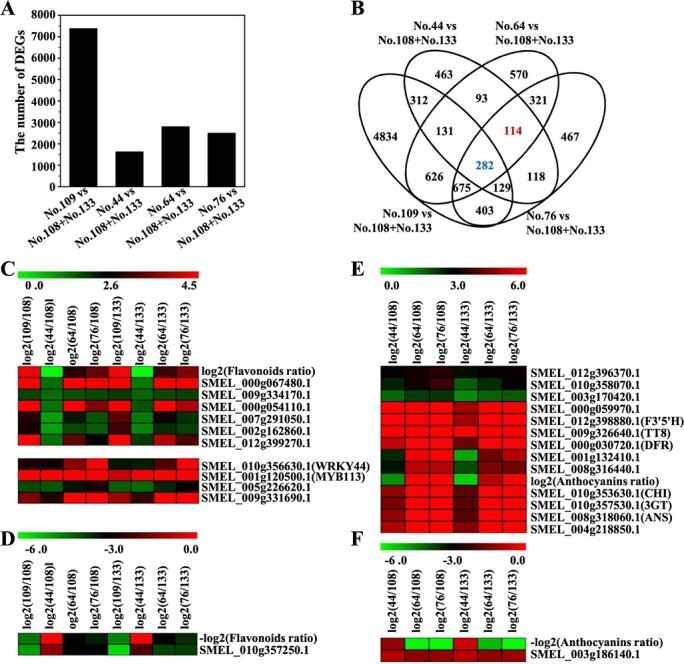
Overview of the DEGs identified among the six eggplant cultivars. (A) The number of DEGs in the No. 109/No. 44/No. 64/No. 76 vs. No. 108 + No. 133 comparison groups. (B) A four-way Venn diagram indicating the DEGs related to flavonoid and anthocyanin biosynthesis. (C) Genes with the same expression patterns as those of *SmMYB113* and *SmWRKY44* that were highly correlated with the flavonoids content. (D) The gene showing the opposite expression pattern but highly correlated with the flavonoids content. (E) Genes showing the same expression pattern that were highly correlated with the anthocyanin content or structural genes. (F) Genes showing opposite expression patterns but highly correlated with the anthocyanin content.

According to the peel color, flavonoids content, and the delphinidins/flavonoids ratio of the six eggplant cultivars reported in our previous study [7], the six eggplant cultivars could be divided into three classes: No. 44, No. 64, and No. 76, which have a purple peel and the highest delphinidin/flavonoids ratio; No. 109, which has an orange peel and the highest content of flavonoids; and No. 108 and No. 133, which have white and green peels, the lowest delphinidins/flavonoids ratio, and the lowest flavonoids content. To isolate flavonoid and anthocyanin biosynthesis related-genes and clarify the molecular mechanism underlying the color formation process in eggplant peels, we compared the fragments per kilobase of transcript per million fragments mapped (FPKM) values of genes in No. 44, No. 64, No. 76, and No. 109 with those in No. 108 and No. 133. First, the DEGs in the No. 108 vs. No. 133 comparison group were identified and abandoned. The remaining genes in the No. 108 and No. 133 (named No. 108 + No. 133) vs. No. 109, No. 44, No. 64, and No. 76 comparison groups were determined, respectively. As shown in Fig. 2A, 7,392, 1642, 2812, and 2509 common DEGs were identified in the No. 109, No. 44, No. 64, and No. 76 vs. No. 108 + No. 133 comparison group, respectively. Venn analysis revealed that 282 and 114 DEGs might be closely related to flavonoid and anthocyanin biosynthesis, respectively (Fig. 2B). Among the 282 DEGs, 129 and 65 DEGs were up-regulated and down-regulated, respectively, in the No. 109, No. 44, No. 64, and No. 76 vs. No. 108 + No. 133 comparison groups. The well-known anthocyanin biosynthesis regulatory genes *SmMYB113* and *WRKY44* (*SmTTG2*) were among the 129 up-regulated DEGs. We then examined the correlations of the 129 up-regulated DEGs and 65 down-regulated DEGs with flavonoid biosynthesis. According to hierarchical clustering (HCL) analysis, six genes were classified with the log_2_^(Flavonoids ratio)^ into one group, but the expression patterns of *SmMYB113* and *WRKY44* were not related to log_2_^(Flavonoids ratio)^ values (Fig. 2C and Supplementary Data Fig. S3A). Two genes showed the same expression patterns and were highly correlated with the expression of *SmMYB113* and *SmWRKY44* (Fig. 2C and Supplementary Data Fig. S3A). Only one of the 65 down-regulated DEGs had the opposite value of log_2_^(Flavonoids ratio)^ (Fig. 2D and Supplementary Data Fig. S3B).

Among the 114 DEGs, 48 and 43 DEGs were commonly up-regulated and down-regulated in No.44, No.64, and No.76 compared with No.108 + No.133, respectively. Strikingly, the anthocyanin biosynthesis structural genes *SmCHI*, *SmF3’5’H*, *SmDFR*, *SmANS*, and *Sm3GT* and the anthocyanin biosynthesis regulatory gene *TT8* were among the 48 up-regulated DEGs. HCL analysis showed that these anthocyanin biosynthesis-related genes and log_2_^(Anthocyanins ratio)^ values were classified into one group, and another seven genes had the same expression patterns and were highly correlated with them (Fig. 2E and Supplementary Data Fig. S3C). As for the 43 down-regulated DEGs, only one gene had the opposite value of log_2_^(Anthocyanins ratio)^ (Fig. 2F and Supplementary Data Fig. S3D).

### Key genes affecting the different purple peel color in eggplant

**Figure 3:**
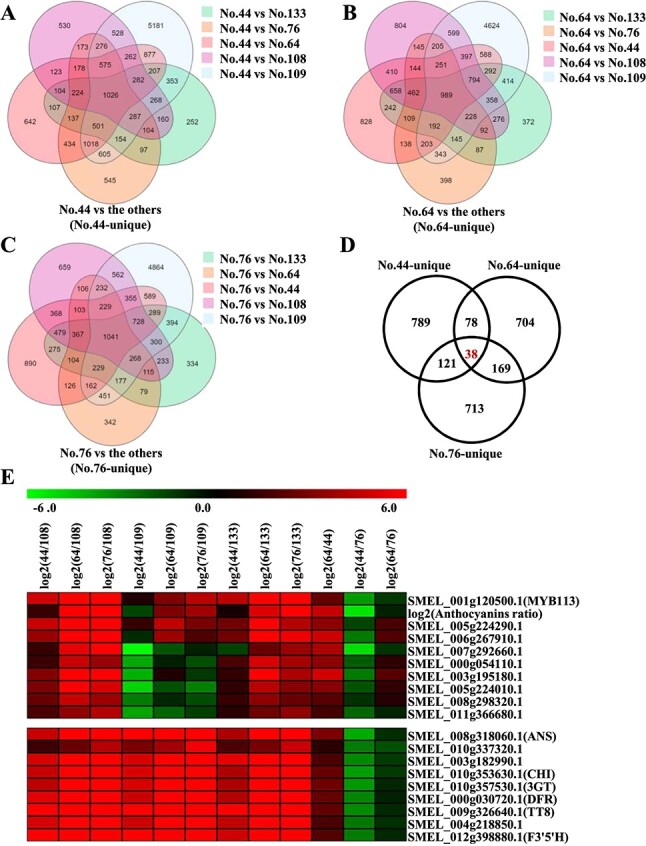
Venn analysis and HCL analysis were used to identify the key genes regulating differences in the anthocyanin content in the peels of different eggplant varieties. (A–C) Five-way Venn diagram indicating the common DEGs in the comparison groups of No. 44/No. 64/No. 76 vs. the other five eggplant cultivars. (D) Venn analysis identified 38 DEGs related to differences in purple-peel color. (E) The genes showing the same expression patterns that were highly correlated with the anthocyanin content or structural genes.

Given that the peel color and the content of anthocyanins of the six eggplant cultivars differed, the common DEGs in the comparison groups of No. 44/No. 64/No. 76 vs. all five of the other eggplant cultivars were analyzed, and these were referred to as No.44-unique, No.64-unique, and No.76-unique, respectively (Fig. 3A–C). Subsequently, Venn analysis revealed 38 genes in the three eggplant cultivars with a purple peel (No. 44, No. 64, and No. 76) that were significantly different from the other three eggplant cultivars that did not have purple color; these genes also significantly differed among No. 44, No. 64, and No. 76 (Fig. 3D). *SmCHI*, *SmF3’5’H*, *SmDFR*, *SmANS*, *Sm3GT*, *SmMYB113*, and *SmTT8* were among the 38 DEGs. HCL analysis showed that *MYB113* and log_2_^(Anthocyanins ratio)^ were classified into one group, and another eight genes had the same expression pattern and were highly correlated with them (Fig. 3E and Supplementary Data Fig. S4). The anthocyanins biosynthesis structural genes and *TT8* were classified into one group with another three genes (Fig. 3E and Supplementary Data Fig. S4).

**Figure 4:**
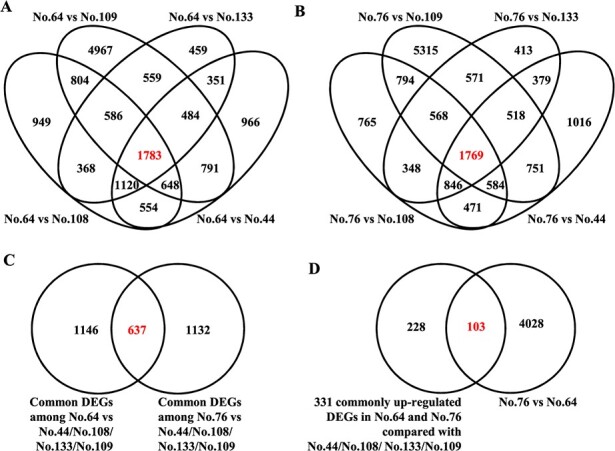
Venn analysis revealed the key genes determining the different purple-peel color in eggplant. (A,B) Four-way Venn diagram indicating the common DEGs in the No. 64/No. 76 vs. No. 44/No. 108/No. 133/No. 109 comparison groups. (C) Venn analysis revealing the common regulated DEGs in the No.64/No.76 vs. No. 44/No. 108/No. 133/No. 109 comparison groups. (D) Venn analysis revealing the common up-regulated DEGs that were differentially expressed between No. 64 and No. 76.

In our previous study, we found that the delphinidins/flavonoids ratio of No. 64 and No. 76 was similar and significantly greater than that of the other four eggplant cultivars; all the anthocyanins detected in No. 64 were also detected in No. 76, and the content of most anthocyanins was lower in No. 64 than in No. 76, with the exception of tulipanin and cyanidin [7]. Therefore, we speculate that the genes related to delphinidin biosynthesis were differentially expressed between No. 64 and No. 76. First, the common DEGs in the comparison groups of No. 64/No. 76 vs. the other four eggplant cultivars were determined (Fig. 4A–B). A total of 637 genes were significantly differentially expressed in the No. 64/No. 76 vs. No. 44/No. 108/No. 133/No. 109 comparison groups (Fig. 4C). Among the 637 DEGs, 331 and 88 genes were commonly up-regulated and down-regulated in No. 64 and No. 76 compared with No. 44/No. 108/No. 133/No. 109.

Among the 331-common-up-regulated genes, 103 genes were significantly differentially expressed between No. 64 and No. 76 (Fig. 4D). The FPKM values of the known anthocyanin biosynthesis genes *SmCHI*, *SmF3H*, *SmF3’5’H*, *SmDFR*, *SmANS*, *Sm3GT*, *SmMYB113*, and *SmTT8* were greater in No. 76 than in No. 64, indicating that anthocyanin biosynthesis was up-regulated to a greater degree in No. 76 than in No. 64; consequently, the anthocyanin content was highest in No. 76. A DEG encoding an acyltransferase enzyme was more highly expressed in No. 64 than in No. 76, indicating that it might play an important role in anthocyanin accumulation.

### Identification of the SmMYB113-regulated anthocyanin biosynthesis network

**Figure 5:**
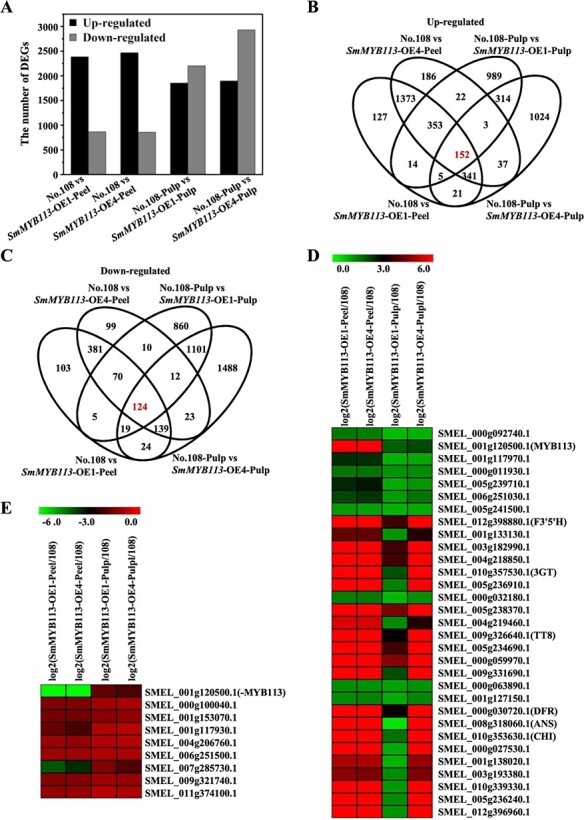
Overview of the DEGs identified from the peel and pulp in the *SmMYB113*-OE1 and OE4 vs. No. 108 comparison groups. **(**A) The number of DEGs in the peel and pulp in the *SmMYB113*-OE1 and OE4 vs. No. 108 comparison groups. (B,C) Four-way Venn diagram revealing the up-regulated (B) and down-regulated (C) DEGs involved in the SmMYB113-regulated network. (D) The genes showing the same expression patterns as those of *SmMYB113* and structural genes. (E) The genes showing opposite expression patterns with that of *SmMYB113*.

The peel and pulp of *SmMYB113*-overexpressing transgenic eggplants (*SmMYB113*-OE1 and *SmMYB113*-OE4) were purple due to the accumulation of anthocyanins, whereas the peel and pulp of No. 108 (WT) were white (Supplementary Data Fig. S1A-B). To identify the genes involved in the SmMYB113-regulated anthocyanin biosynthesis network, the peel and pulp of *SmMYB113*-OE1 and OE4 eggplant lines were also transcriptome analyzed with the above six eggplant cultivars at the same time (Supplementary Data Table S2). The FPKM values of the genes in the peel and pulp of the *SmMYB113*-OE1 and OE4 eggplant lines were compared with those in No. 108. A total of 3251 (2386 up-regulated; 865 down-regulated), 3325 (2467 up-regulated; 858 down-regulated), 4053 (1852 up-regulated; 2201 down-regulated), and 4827 (1897 up-regulated; 2930 down-regulated) DEGs were detected between the peel and pulp in the *SmMYB113*-OE1 and OE4 vs. No. 108 comparison groups (Fig. 5A). Among these DEGs, 152 genes were up-regulated, and 124 genes were down-regulated simultaneously in the peel and pulp of both *SmMYB113*-overexpressing lines compared with No. 108 (Fig. 5B–C). HCL analysis revealed that the expression of *SmMYB113*, *SmTT8*, and the anthocyanin biosynthesis structural genes was up-regulated; these genes were classified into one group, and another 24 genes had the same expression pattern and were highly correlated with them (Fig. 5D and Supplementary Data Fig. S5A). Among the 124 down-regulated genes, eight genes had the same expression pattern and were highly correlated with the opposite log_2_^(sample1/sample2)^ value of *MYB113* (Fig. 5E and Supplementary Data Fig. S5B).

### GO and KEGG enrichment analysis of the detected novel genes

**Figure 6:**
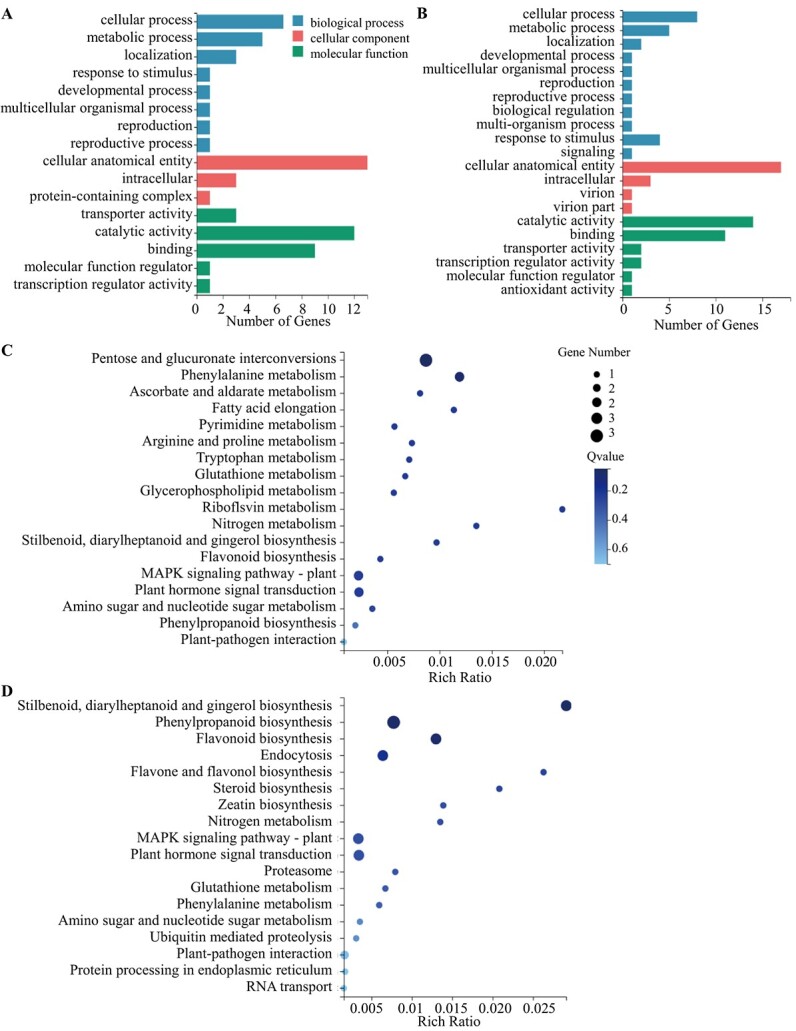
Results of the GO and KEGG analyses of the 27 and 32 novel genes associated with differences in peel color among eggplant peels and involved in the SmMYB113-regulated anthocyanin biosynthesis network, respectively. (A,B) Results of the GO analysis of the 27 (A) and 32 (B) novel genes. (C,D) Results of the KEGG enrichment analysis of the 27 (C) and 32 (D) novel genes.

According to the above analytical strategies, a total of 27 novel genes were identified to be related to color differences among eggplant peels, and 32 novel genes played a role in the anthocyanin biosynthesis network regulated by SmMYB113 (Supplementary Data Table S3). To understand the biological function of these 27 and 32 novel genes, GO and KEGG analysis were carried out. As shown in Fig. 6A and 6B, the 27 and 32 novel genes were most significantly enriched in the following GO terms: “cellular process” and “metabolic process” in the biological process category; “cellular anatomical entity” in the cellular component category; and “catalytic activity” and “binding” in the molecular function category. The results of the KEGG pathway analysis are shown in Fig. 6C and 6D. These 27 and 32 novel genes were enriched in 10 pathways, including “Phenylpropanoid biosynthesis,” “Flavonoid biosynthesis,” “Nitrogen metabolism,” “Glutathione metabolism,” “Phenylalanine metabolism,” “Amino sugar and nucleotide sugar metabolism,” “MAPK signaling pathway – plant,” “Plant hormone signal transduction,” and “Plant-pathogen interaction.”

### Functional characterization of the five novel genes involved in anthocyanin biosynthesis

**Figure 7:**
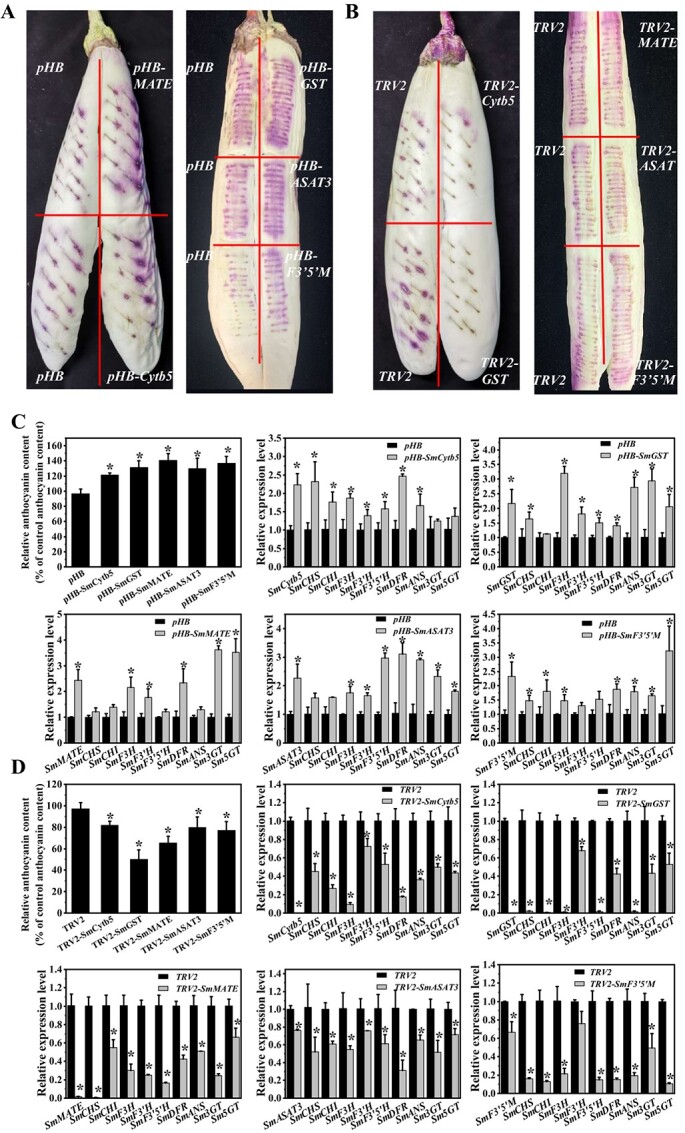
Functional characterization of the five novel genes in anthocyanin biosynthesis. (A,B**)** Transient expression assay in eggplant peels with the overexpression vector (A) and virus-induced gene silencing vector (B). (C,D) The relative anthocyanin content and expression level of anthocyanin biosynthesis structural genes in eggplant peels infiltrated with the overexpression vector (C) and virus-induced gene silencing vector (D). Data are means ± SD (*n* = 3 biological replicates). ^*^P < 0.05

Venn analysis revealed five genes that were shared among both sets of novel genes, and they were *SmCytb5*, *SmGST*, *SmMATE*, *SmASAT3*, and *SmF3’5’M* (Supplementary Data Fig. S6A). To identify the role of the five novel genes, a tobacco leaf transient expression assay was carried out. The results showed that these genes could not induce anthocyanin biosynthesis in tobacco leaves alone (Supplementary Data Fig. S6B). The eggplant fruits grown without light were used for another transient expression assay. *SmMYB113* was expressed and it mediated the synthesis of anthocyanins following exposure to light. The coloration, anthocyanin content and the relative expression level of anthocyanin biosynthesis structural genes were enhanced via the infiltration of the *pHB*-*SmASAT3*, *SmGST*, *SmMATE*, *SmCytb5*, or *SmF3’5’M* vector compared with the empty vector *pHB* (Fig. 7A and 7C).

The virus-induced gene silencing system was used to further validate the functions of the five genes. Reduced coloration in the peel around the injection sites was observed after the five genes were silenced (Fig. 7B). The anthocyanin content and the relative expression level of anthocyanin biosynthesis structural genes in the peel around the injection sites of pTRV1/pTRV2-*SmCytb5*, pTRV2-*SmGST*, pTRV2-*SmMATE*, pTRV2-*SmASAT3*, and pTRV2-*SmF3’5’M* was significantly decreased compared with that in the injected sites in the corresponding controls (Fig. 7D). These results suggest that these five genes play important roles in anthocyanin accumulation in eggplant.

### Analysis of the relationships between SmMYB113 and the promoters of the five genes

**Figure 8:**
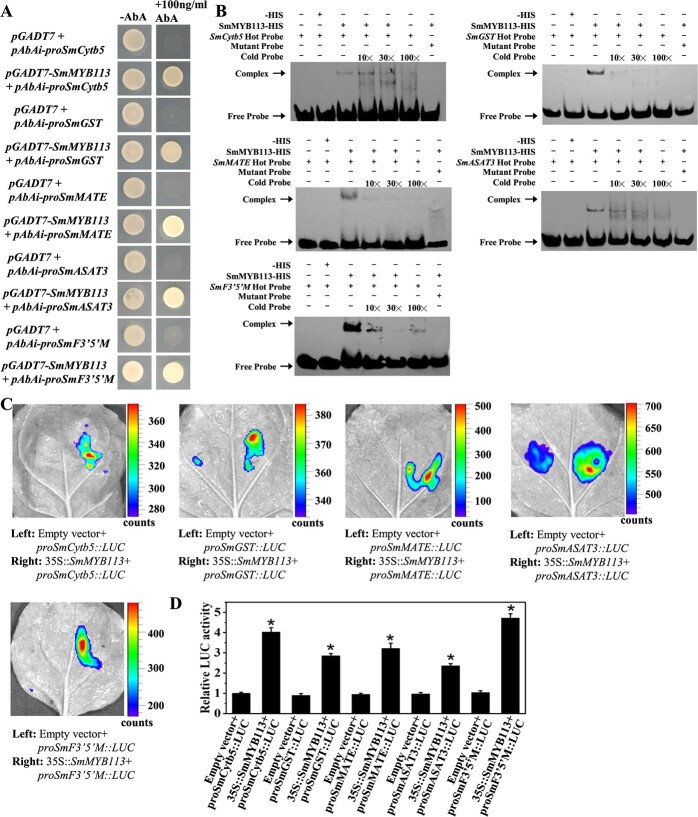
SmMYB113 binds to the promoter of the five genes and promotes their expression. (A) Y1H assay. The positive clones were identified on SD-Leu-Ura medium with or without AbA (100 ng ml^−1^). (B) EMSA. Hot probe represents the fragment with a biotin label. Mutant probes were produced by replacing the MYB-binding cis-elements. Excess cold, unlabeled probes were used as competitors. + and – represent presence and absence, respectively. 10×, 30× and 100 × represent the relative concentration of competition probe. (C,D) Transcriptional activation of SmMYB113 on the promoters of the five genes was confirmed by a dual-luciferase assay in tobacco leaves. Data are means ± SD (*n* = 3 biological replicates). ^*^ P < 0.05.

The expression levels of the five genes were all increased when *SmMYB113* was overexpressed. In addition, some MYB binding cis-elements were found in all the promoters of the five genes. Therefore, a series of biochemical experiments were performed to study the interaction of SmMYB113 and the promoters of the five genes. First, Y1H assay showed that Y1H Gold yeast cells co-transformed with *pGADT7-SmMYB113* and *pAbAi-proSmCytb5*, *pAbAi-proSmGST*, *pAbAi-proSmMATE*, *pAbAi*-*proSmASAT3*, and *pAbAi-proSmF3’5’M* could all survive on the selective medium (lacking Leu and Ura and containing 100 ng/ml AbA), whereas those containing the negative control could not survive on the selective medium (Fig. 8A). Then, electrophoretic mobility shift assay (EMSA) results showed that SmMYB113 protein could bind to the probe with the MYB binding cis-elements from the five genes and caused a mobility shift, while the signal of the shifted band was weakened by the increased competition probe (Fig. 8B). These data indicate that SmMYB113 can bind to the promoters of the five genes.

A dual-luciferase assay was performed to identify the regulation relationship between SmMYB113 and the five genes in vivo. The *35S*::*SmMYB113* vector was co-infiltrated into tobacco leaves with the construct *proSmCytb5:LUC*, *proSmGST*:*LUC*, *proSmMATE*:*LUC*, *proSmASAT3*:*LUC*, or *proSmF3’5’M*:*LUC*. The luminescence intensity and relative LUC activity were stronger in tissues co-expressing *35S*::*SmMYB113* and *proSmCytb5:LUC*, *proSmGST*:*LUC*, *proSmMATE*:*LUC*, *proSmASAT3*:*LUC*, or *proSmF3’5’M*:*LUC* than that in the control (Fig. 8C-D). These results indicate that SmMYB113 activates the expression of these five genes in eggplant.

## Discussion

Unlike previously published studies that focused on clarifying the molecular mechanisms of the color formation process induced by light in eggplant peels [9, 10, 17], we compared six eggplant cultivars with different peel color at the transcriptional level to explore the molecular mechanism of the different color formation among eggplant peels in this study. Moreover, the regulatory network of SmMYB113 in anthocyanins biosynthesis was firstly genome-wide revealed.

Two analytical strategies were used to identify the genes related to different peel color formation in eggplants, and both analytical strategies revealed previously identified anthocyanin biosynthesis structural genes and anthocyanin biosynthesis regulatory genes. This suggests that the results of these two analytical strategies were robust. The functions of the 27 novel genes in eggplant have not yet been reported, but some of their homologous genes from other plant species have been reported to play a role in anthocyanin biosynthesis, such as *MdERF38* [18] and *PpGST1* [19]. These results further suggest that the identified 27 novel genes were highly correlated with the color differences among the peels of the six eggplant cultivars. Because the relative flavonoids content was highest and the anthocyanins/flavonoids ratio was low in No. 109, the color of its peel was orange [7]. When we regarded No.109, No.44, No.64 and No.76 as one category comparing with No.108 and No.133, the expression level of *SmMYB113* and *SmWRKY44* differed in the two categories (Fig. 2 and Supplementary Data Fig. S2). However, no correlation was observed in the expression of *SmMYB113* and *SmWRKY44* with the relative flavonoids content in the six eggplant cultivars. Based on the reported function of *SmMYB113* and *SmWRKY44* in eggplant [7, 20], SmWRKY44 might interact with SmMYB113 to promote anthocyanin biosynthesis, and the overexpression of *SmMYB113* might also increase the content of other flavonoids. These findings suggest that *SmMYB113* and *SmWRKY44* might play a role in flavonoid biosynthesis, but they are not key regulators. Similarly, the interaction between SmTT8 and SmMYB113 might only contribute to anthocyanin biosynthesis, which corresponds to the reduced PAs and anthocyanins phenotype of *mttt8* mutant [21].

MYB family members comprise one of the largest transcription factor families in plants, and they are involved in various physiological and biochemical processes in plants, such as phenylpropanoid metabolism and the abiotic stress response [22, 23]. In eggplant, the role of SmMYB113 in anthocyanin biosynthesis has been clarified [7, 9, 14], while the other roles were still unknown. Here, 32 novel genes showed the same expression patterns and were highly correlated with the expression of *SmMYB113* (Fig. 5). GO and KEGG analysis suggested that SmMYB113 might play roles in plant hormone signal transduction and other metabolic pathways (Fig. 6). Moreover, *SmMYB113*-OE eggplant lines were more cold-tolerant than WT plants (data not shown). A previous study has reported that the interaction of SmCBFs with SmMYB113 can enhance SmMYB113 regulatory effects, resulting in the increase of anthocyanin accumulation at low temperatures [15]. Therefore, the underlying molecular mechanism of SmMYB113 in elevating cold-tolerance was worthy to reveal in the future, including whether it is mediated by the interaction between SmMYB113 and SmCBFs or other pathways.

Here, five genes, *SmCytb5*, *SmGST*, *SmMATE*, *SmASAT3*, and *SmF3’5’M*, were closely related to color differences among eggplant peels, and their expression was directly regulated by SmMYB113 (Fig. 7–8). Cytb5 has been reported to be required for the activity of F3’5’H, which affects the accumulation of anthocyanins and flower color [24, 25]. In this study, the expression of *SmCytb5* was associated with *SmF3’5’H* and peel color (Fig. 3), which is consistent with the results of previous studies of the skin of red grapes [26], blood orange [27], and black-colored jaboticaba peels [28]. The results of our previous study indicate that delphinidins are the main class of anthocyanins in eggplant cultivars with purple peels and in the peel and pulp of *SmMYB113*-OEs [7], which might be explained by the expression level of *SmF3’5’H*. However, we were unable to characterize the transcriptional regulatory relationship between SmMYB113 and the promoter of *SmF3’5’H* because of the high self-activation activity of the *SmF3’5’H* promoter, which has also been observed in kiwifruit [29]. However, there was a clear transcriptional regulatory relationship between SmMYB113 and the promoter of *SmCytb5*. Therefore, we speculate that SmMYB113 might regulate the activity of F3’5’H by enhancing *SmCytb5* expression, which results in different purple-peels color among eggplants. Previous studies have revealed three ways in which anthocyanins can be transported in plants: GST-mediated transport, MATE-type anthocyanin transporters, and membrane vesicle-mediated transport [30–33]. Therefore, we speculate that SmMYB113 might enhance anthocyanin biosynthesis in the cytoplasm and promote the transport of anthocyanins to the vacuole by enhancing the expression of *SmGST* and *SmMATE*. A previous study has reported that a regiospecific flavonoid, 3’5’-O-methyltransferase (*F3’5’M*), could efficiently convert quercetin, luteolin, and eriodictyol to isorhamnetin, chrysoeriol, and homoeriodictyol, respectively [34]. However, the roles of the *F3’5’M* in the anthocyanin biosynthesis pathway have not yet been clarified. Previous studies have reported that a BAHD anthocyanin acyltransferase (SmelAAT) controls the conversion of delphinidin-3-rutinoside to nasunin, whereas the acyltransferase, sinapoyl-Glc: anthocyanin acyltransferase (SAT) enhances Cy3XSGG production in carrot [35, 36]. In our study, the *SmF3’5’M* was highly expressed in No. 76, followed by No. 64, and the acylsugar acyltransferase enzyme (*SmASAT3*) was highly expressed in No. 64, followed by No. 76. Functional analysis results indicated that *SmF3’5’M* and *SmASAT3* promoted anthocyanin accumulation in eggplant, and the expression of both genes was directly regulated by SmMYB113 (Fig. 7–8). Therefore, *SmF3’5’M* and *SmASAT3* might play important roles in modifying anthocyanin substrates in eggplant, resulting in differences in purple color.

## Conclusion

**Figure 9:**
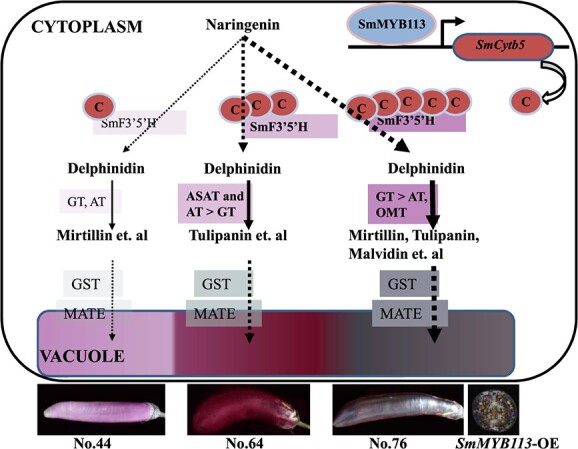
A regulatory model for differences in color formation in eggplant peels. SmMYB113 regulates the activity of SmF3’5’H, which is critical for shaping variation in purple peels, by enhancing *SmCytb5* expression. The acylation (ASAT and AT)/glycosylation (GT) ratio of delphinidins determines the reddish-purple and black-purple peel color. The activity of GST and MATE, which are responsible for anthocyanin transport, is also regulated by SmMYB113.

The aim of this study was to clarify the molecular mechanism of the different colors among eggplant peels and reveal the SmMYB113-regulated anthocyanin biosynthesis regulatory network. RNA-seq analysis was used to identify novel genes involved in flavonoid and anthocyanin biosynthesis in eggplants. Five novel genes were identified to be related to differences in the color of the peel and play a role in the SmMYB113-regulated anthocyanin biosynthesis network at the transcriptional level. The relationships among these five genes, anthocyanin biosynthesis, and SmMYB113 were analyzed in eggplant for the first time. Finally, a regulatory model was proposed (Fig. 9). SmMYB113 is a key transcription factor that affects the content and structure of anthocyanins. SmMYB113 might regulated the activity of SmF3’5’H that were critical for the variation in purple peels by enhancing the *SmCytb5* expression. The acylation (ASAT and AT)/glycosylation (GT) ratio of delphinidins determined the reddish-purple and black-purple color of eggplant peel. More studies are needed to clarify the role of delphinidin methylation in purple color formation. The activity of SmGST and SmMATE, which mediate anthocyanin transport, was also regulated by SmMYB113. Our results enhance our understanding of how the color of the peel of eggplant is determined; our findings will aid subsequent studies of flavonoids, anthocyanin biosynthesis, and molecular breeding.

## Materials and methods

### Plant material

The six eggplant cultivars and *SmMYB113*-overexpression (OE) transgenic eggplants used for sampling were the same as those used in our previous study [7], and the peels or pulps of the fruits at the rapid growth (G) stage were used for RNA extraction. Three biological replicates were conducted for each sample.

### RNA extraction, mRNA library construction, and sequencing

The TaKaRa MiniBEST Plant RNA Extraction Kit (TaKaRa, Otsu, Shiga, Japan) was used to extract total RNA from samples. The RNA quality was determined using a Nanodrop 2000 spectrophotometer (Thermo Scientific, Wilmington, Delaware), 1.2% agarose gel electrophoresis, and an Agilent 2100 Bioanalyzer. The mRNA was enriched using oligo (dT) magnetic beads, and rRNA was removed using DNA probes and RNaseH. The purified and fragmented mRNA was then synthesized to double-stranded cDNA (dscDNA) using N6 random primers. The 5′ and 3′ terminals of the dscDNA were phosphorylated and adenylated, respectively. The ligation products were amplified using polymerase chain reaction (PCR) primers and then denatured by heat. The single-stranded DNA was cyclized for library construction. The libraries were sequenced using the DNBSEQ platform, and raw reads were produced.

### Bioinformatics analysis

The obtained raw reads were subjected to quality control analysis to remove reads with low quality. The clean reads were then assembled into unigenes and mapped to the eggplant genome sequences [16] (https://solgenomics.net/organism/Solanum_melongena/genome) using the HISAT program [37]. The gene expression levels were calculated using the RSEM package [38]. The DESeq2 package in R software was used to identify DEGs. The identified DEGs were annotated via comparison with the Gene Ontology (GO), National Center for Biotechnology Information non-redundant, and Kyoto Encyclopedia of Genes and Genomes (KEGG) databases. Multi-Experiment Viewer software with a color scale (MeV v4.4.1, http://www.tm4.org/) was used to generate cluster diagrams via the hierarchical clustering method [39].

### qRT-PCR

The qRT-PCR assay was done using methods detailed in [7]. The *actin* gene (*SmACT7*) was used as an internal reference to normalize the expression levels via the 2^-△△Ct^ method [40].

### Y1H

The promoter fragments of *SmCytb5*, *SmGST*, *SmMATE*, *SmASAT3*, and *SmF3’5’M* were constructed into the *pAbAi* vector as bait, respectively. The coding sequence (CDS) of *SmMYB113* was constructed into the *pGADT7* vector as prey. The bait and prey vector were transformed into the yeast strain Y1H Gold sequentially. The positive clones were identified on SD-Leu-Ura medium with or without AbA (100 ng ml^−1^).

### Electrophoretic mobility shift assay

The SmMYB113-pET32a recombinant plasmid was transformed into *Escherichia coli* BL21 cells for purification of the His-tagged fusion protein. All probes (biotin-labeled, mutated probes and unlabeled) were synthesized by Sangon Biotech (Shanghai, China). The experiment was carried out according to the manufacturer’s instructions (www.thermoscientific.com/pierce).

### Dual-luciferase assay

The promoter fragments of *SmCytb5*, *SmGST*, *SmMATE*, *SmASAT3*, and *SmF3’5’M* were inserted into *pGreenII 0800-LUC* vector, and the CDS of *SmMYB113* was inserted into an overexpression vector *pHB*. The recombinant plasmids were individually introduced into *Agrobacterium strain* GV3101. 10 mM MES, 10 mM MgCl_2_, and 100 μM AS were mixed with MS (pH 5.8) used as infiltration buffer. The infiltration buffer with *Agrobacterium* was adjusted to an OD_600 nm_ of 0.6. The luminescence intensity was detected following a previously described method [41], and the renilla luciferase and firefly luciferase activities were tested by the Dual-Luciferase® Reporter Assay System (E1910, Promega, USA). This experiment was repeated 3 times.

### Transient expression assay in tobacco leaves and eggplant fruits

The CDSs of *SmMYB113*, *SmCytb5*, *SmGST*, *SmMATE*, *SmASAT3*, and *SmF3’5’M* were constructed into an overexpression vector *pHB*, respectively. The *Agrobacterium* infiltration buffer containing *pHB***-***SmMYB113*, *-SmCytb5*, -*SmGST*, -*SmMATE*, *-SmASAT3*, and -*SmF3’5’M* or empty vector was injected into tobacco leaves. The purple color in tobacco leaves was observed after inoculation for 4 days.

The gene-specific primers were designed using the SGN VIGS Tool to specifically silence the *SmCytb5*, *SmGST*, *SmMATE*, *SmASAT3*, and *SmF3’5’M* genes (https://vigs.solgenomics.net/). Next, 300-bp fragments were constructed into the *TRV2* vector. The *Agrobacterium* infiltration buffer containing *pHB***-***SmCytb5*, **-***SmGST*, **-***SmMATE*, *-SmASAT3*, and **-***SmF3’5’M* or empty vector, and *TRV1* mixed with *TRV2-SmCytb5*, **-***SmGST*, **-***SmMATE*, *-SmASAT3*, and **-***SmF3’5’M* or empty vector were injected into the following eggplant peels. The purple peel cultivar “Dan Hong” was used, of which anthocyanin biosynthesis depended on light. The eggplant flowers were covered with black paper bags for 25 days after full bloom and white peel fruits were obtained. The infiltrated eggplant fruits were treated under darkness overnight and moved to low light condition (60 μmol·m^−2^·s^−1^). The stalk of eggplant fruits was covered with a moist cotton ball and wrapped with plastic wrap to keep them fresh. The color of eggplant peels and their anthocyanin content were measured after inoculation for 3 days. Three biological replicates were conducted for each gene in the experiments and repeated 3 times.

### Extraction and quantification of anthocyanins

Anthocyanins were extracted and the content of anthocyanins was quantified following the methods described in a previous study [42].

### Statistical analysis

Data were expressed as mean ± standard deviation. Statistical analysis was conducted using SPSS 17.0 (SPSS, Inc., Chicago, USA) software, and Duncan’s new multiple range test was used to evaluate the significance of mean differences between treatments. The threshold for statistical significance was *P*-value <0.05.

All the primers used in this study are listed in Supplementary Data Table S1.

## Supplementary Material

Web_Material_uhad181
