# Supplementary material for: RNA-sequencing analysis reveals novel genes involved in the different peel color formation in eggplant
Source: Hortic Res. 2023 Sep 4;10(10):uhad181. doi: 10.1093/hr/uhad181 (PMC10599318; doi:10.1093/hr/uhad181)
Supplement: Web_Material_uhad181 [file web_material_uhad181.zip › Supplementary Tables.docx]

**Table S1 List of primer sequences used in this study.**

| **Gene** | **Forward primer (5’-3’)** | **Reverse primer (5’-3’)** |
| --- | --- | --- |
| **For real-time qPCR** | | |
| *Smactin* | GTCGGAATGGGACAGAAGGATG | GTGCCTCAGTCAGGAGAACAGGGT |
| *SmCHS* | GGGAACAGTACTCCGGCTAGCC | AACACCTGAAATTGGGTCTGAACCA |
| *SmCHI* | CCTTGACGGGTAAGCAATACTCTG | GATGGAGGCACCATGTGGGAAGG |
| *SmF3H* | GTGGTCCAAGACTGGCGTGAAAT | TTCTCTAACCCCATTGCTTCTGATA |
| *SmF3’H* | CCTGTTGCACCCTTGTTGATTC | CGGGATCCTTTGGGTATGTGA |
| *SmF3’5’H* | TGGACCTCGTTGGAAGTTGCTAAG | TGCCATCGCGAACGTCAACATAT |
| *SmDFR* | GGCCATTGAGACTTGCCGACAG | CACCATTGGTCAACTGTCCTGTACT |
| *SmANS* | CTCGATTCCCACCTCGGACCTT | TCAGCTGCAGCGTCCTGTTTGT |
| *Sm3GT* | GCATCCAATGGCTTGACAAACA | TTCTGCTATCGACCCAATTTCG |
| *Sm5GT* | ATGTGTGGAAGAGTGGAGTTAGAG | TCCCCTCCATCCATTACCA |
| *SmMYB113* | AAAGTTGTAGGCTGAGGTGGTTGA | GAGATCCACTTCATCCGAAGCA |
| *SmWRKY44* | ACCCTGCTGTCGGAAATTCTG | TGCTCTGTTGGACAGGCCTTT |
| *SmTT8* | TTCTGCCGGAGACTCAAATCAG | AAACCATTCGGATTCCGTCAAG |
| **For** **transient expression assay** | | |
| *pHB-SmASAT3* | TCTCTCTCTCAAGCTTATGGCATCATCAAGAATTATGTCTAGA | TGCAGCTCGAGGATCCTTATTCCGATGACCAACCAACC |
| *pHB-SmGST* | TCTCTCTCTCAAGCTTATGGTAGTGAAAGTGTATGGTTCAGC | TGCAGCTCGAGGATCCTTAAAGTGTTACTTCTTCTTTAGCTGGC |
| *pHB-SmMATE* | TCTCTCTCTCAAGCTTATGGAGACGCCGTTGCTGA | TGCAGCTCGAGGATCCCTACACCTCCATTCTCATAGCTAACTG |
| *pHB-SmF3’5’M* | TCTCTCTCTCAAGCTTATGTACATTTTGGAAAGTAGTGTTTATCC | TGCAGCTCGAGGATCCCTAGTCGAGACGCCGGCA |
| *pHB-SmCytb5* | TCTCTCTCTCAAGCTTATGGACACAAAAAATATGTTCACCC | TGCAGCTCGAGGATCCTCAAATCTGGAGAGCTCCAGTGG |
| *pHB-SmMYB113* | TCTCTCTCTCAAGCTTATGAATAATCCTCCTATAATCTGTACGTC | TGCAGCTCGAGGATCCTTAATCAAGTAGATTCCATAAATCAATATCA |
| *TRV2-SmASAT3* | TAAGGTTACCGAATTCGCTTGGGAAGACTCAAGGCC | GCTCGGTACCGGATCCCCCGAAGTTGTTGTTTTGCC |
| *TRV2-SmGST* | TAAGGTTACCGAATTCATGGTAGTGAAAGTGTATGGTTCAGC | GCTCGGTACCGGATCCGGTCAAGTCACTGATCTGTCCCA |
| *TRV2-SmMATE* | TAAGGTTACCGAATTCATGGAGACGCCGTTGCTGA | GCTCGGTACCGGATCCGGCACTTCCCATTCCCATCA |
| *TRV2-SmF3’5’M* | TAAGGTTACCGAATTCGACAAAGATGCATATGAAGTTGGATT | GCTCGGTACCGGATCCCAATGCCTTTAACCCTGGTGG |
| *TRV2-SmCytb5* | TAAGGTTACCGAATTCGTAATAGACGTAACGAAGTTTCTGGAA | GCTCGGTACCGGATCCGAAAGGAACAAAATACTCAACAAAAAC |
| **For Y1H assay** | | |
| *pGADT7-SmMYB113* | GGAGGCCAGTGAATTCATGAATAATCCTCCTATAATCTGTACGTC | ATGGATCCCGTATCGATATCAAGTAGATTCCATAAATCAATATCA |
| *pAbAi-proSmASAT3* | CCGGGGATCTGTCGACTCAAATAGCCACTGAGTTGCTACAA | GAGCACATGCCTCGAGGACCTCTCAAATATTAATACAAAATATTGAA |
| *pAbAi-proSmGST* | CCGGGGATCTGTCGACTAAAATGTAATACAACAAATAAGATTCTTAACG | GAGCACATGCCTCGAGTTTTTTTTTCTTTTTCTGTGAAATCC |
| *pAbAi-proSmMATE* | CCGGGGATCTGTCGACGATAATGATAAGAAAGAAATGCGTTTTA | GAGCACATGCCTCGAGTCTCAAGTAAAATCTGCTGCACAAT |
| *pAbAi-proSmF3’5’M* | CCGGGGATCTGTCGACGATGAAGTTTTAGTGAAGTTTCATCATG | GAGCACATGCCTCGAGGATGATTCCATAAGGCCTCCTTT |
| *pAbAi-proSmCytb5* | CCGGGGATCTGTCGACATTGGCTAACCTGATGTATGATTTCT | GAGCACATGCCTCGAGATTTTGGGGCATGAAATAGAGAGT |
| **For dual-luciferase assay** | | |
| *proSmASAT3* | CGGTATCGATAAGCTTACTCAAATAGCCACTGAGTTGCTACAA | TAGAACTAGTGGATCCGACCTCTCAAATATTAATACAAAATATTGAA |
| *proSmGST* | CGGTATCGATAAGCTTACTAAAATGTAATACAACAAATAAGATTCTTAACG | TAGAACTAGTGGATCCTTTTTTTTTCTTTTTCTGTGAAATCC |
| *proSmMATE* | CGGTATCGATAAGCTTACGATAATGATAAGAAAGAAATGCGTTTTA | TAGAACTAGTGGATCCTCTCAAGTAAAATCTGCTGCACAAT |
| *proSmF3’5’M* | CGGTATCGATAAGCTTACGATGAAGTTTTAGTGAAGTTTCATCATG | TAGAACTAGTGGATCCGATGATTCCATAAGGCCTCCTTT |
| *proSmCytb5* | CGGTATCGATAAGCTTACATTGGCTAACCTGATGTATGATTTCT | TAGAACTAGTGGATCCATTTTGGGGCATGAAATAGAGAGT |
| **For EMSA assay** |  |  |
| *SmASAT3* Hot Probe | TTGGTTTTTATGGGCACAGTTGCTACTATGGGTGAATA | TATTCACCCATAGTAGCAACTGTGCCCATAAAAACCAA |
| *SmASAT3* Mutant Probe | TTGGTTTTTATGGGCAACTGGTCTACTATGGGTGAATA | TATTCACCCATAGTAGACCAGTTGCCCATAAAAACCAA |
| *SmGST* Hot Probe | CAGACTTTCCACTCAACCACTGGTTGCTGCTCATCTTCA | TGAAGATGAGCAGCAACCAGTGGTTGAGTGGAAAGTCTG |
| *SmGST* Mutant Probe | CAGACTTTCCACTACCAACCGTTGGTCTGCTCATCTTCA | TGAAGATGAGCAGACCAACGGTTGGTAGTGGAAAGTCTG |
| *SmMATE* Hot Probe | AACAATTGCTATAACTACAGTTAGGGAATGCAATTACCG | CGGTAATTGCATTCCCTAACTGTAGTTATAGCAATTGTT |
| *SmMATE* Mutant Probe | AACAATTGCTATAACTATCTGGCGGGAATGCAATTACCG | CGGTAATTGCATTCAAGCCAGAGCTTTATAGCAATTGTT |
| *SmF3’5’M* Hot Probe | TTTTTTTTTTGCTTAGACAACTGGCAAGGATTTTACTAA | TTAGTAAAATCCTTGCCAGTTGTCTAAGCAAAAAAAAAA |
| *SmF3’5’M* Mutant Probe | TTTTTTTTTTGCTTAGAACCAGTGCAAGGATTTTACTAA | TTAGTAAAATCCTTGCACTGGTTCTAAGCAAAAAAAAAA |
| *SmCytb5* Hot Probe | TCCAAGTTTTTTTCTTTGGTTGATAATTTTATCTAACG | CGTTAGATAAAATTATCAACCAAAGAAAAAAACTTGGA |
| *SmCytb5* Mutant Probe | TCCAAGTTTTTTTCTTGAAGGAATAATTTTATCTAACG | CGTTAGATAAAATTATTCCTTCAAGAAAAAAACTTGGA |

**Table S2 Overview of the RNA-sequencing reads generated from each sample.**

| Sample | Total Raw Reads (M) | Total Clean Reads (M) | Total Clean Bases(Gb) | Clean Reads Q20(%) | Clean Reads Q30(%) | Clean Reads Ratio(%) | Total Mapping to genome(%) | Total Mapping to gene (%) |
| --- | --- | --- | --- | --- | --- | --- | --- | --- |
| No.108-1 | 21.75 | 21.62 | 1.08 | 98.07 | 94.4 | 99.42 | 85.61 | 72.51 |
| No.108-2 | 21.75 | 21.66 | 1.08 | 97.62 | 93.32 | 99.59 | 85.16 | 72.29 |
| No.108-3 | 20.42 | 20.33 | 1.02 | 97.48 | 92.93 | 99.59 | 85.07 | 71.9 |
| No.109-1 | 21.75 | 21.66 | 1.08 | 97.65 | 93.41 | 99.6 | 74.42 | 68.38 |
| No.109-2 | 21.75 | 21.66 | 1.08 | 97.81 | 93.85 | 99.6 | 74.61 | 68.61 |
| No.109-3 | 21.75 | 21.66 | 1.08 | 97.73 | 93.64 | 99.6 | 74.34 | 68.47 |
| No.133-1 | 21.75 | 21.62 | 1.08 | 98.08 | 94.47 | 99.39 | 84.75 | 72.78 |
| No.133-2 | 21.75 | 21.66 | 1.08 | 97.7 | 93.56 | 99.59 | 84.42 | 72.7 |
| No.133-3 | 21.75 | 21.61 | 1.08 | 97.8 | 93.77 | 99.35 | 84.58 | 72.27 |
| No.44-1 | 21.75 | 21.68 | 1.08 | 98.28 | 94.72 | 99.7 | 85.42 | 71.11 |
| No.44-2 | 21.75 | 21.62 | 1.08 | 98.22 | 94.85 | 99.42 | 85.16 | 71.8 |
| No.44-3 | 21.75 | 21.61 | 1.08 | 97.95 | 94.13 | 99.36 | 84.99 | 71.28 |
| No.64-1 | 21.75 | 21.63 | 1.08 | 97.71 | 93.47 | 99.47 | 84.78 | 72.66 |
| No.64-2 | 21.75 | 21.63 | 1.08 | 97.85 | 93.89 | 99.46 | 84.81 | 72.97 |
| No.64-3 | 21.75 | 21.63 | 1.08 | 97.66 | 93.41 | 99.48 | 84.55 | 72.5 |
| No.76-1 | 21.75 | 21.61 | 1.08 | 97.81 | 93.76 | 99.35 | 84.34 | 73.26 |
| No.76-2 | 21.75 | 21.62 | 1.08 | 97.88 | 93.9 | 99.39 | 84.59 | 73.46 |
| No.76-3 | 21.75 | 21.61 | 1.08 | 97.64 | 93.28 | 99.35 | 84.39 | 73.62 |
| No.108-Pulp1 | 21.75 | 21.66 | 1.08 | 97.66 | 93.45 | 99.58 | 84.26 | 70.82 |
| No.108-Pulp2 | 21.75 | 21.63 | 1.08 | 98.14 | 94.52 | 99.46 | 84.62 | 70.29 |
| No.108-Pulp3 | 21.75 | 21.66 | 1.08 | 97.57 | 93.27 | 99.57 | 84.06 | 70.43 |
| *SmMYB113*-OE1-Peel1 | 21.75 | 21.67 | 1.08 | 97.63 | 93.34 | 99.63 | 85.04 | 71.69 |
| *SmMYB113*-OE1-Peel2 | 21.75 | 21.67 | 1.08 | 97.79 | 93.78 | 99.63 | 85.21 | 71.92 |
| *SmMYB113*-OE1-Peel3 | 21.75 | 21.66 | 1.08 | 97.79 | 93.79 | 99.62 | 85.17 | 72 |
| *SmMYB113*-OE1-Pulp1 | 21.75 | 21.6 | 1.08 | 97.6 | 93.2 | 99.33 | 84.19 | 69.56 |
| *SmMYB113*-OE1-Pulp2 | 21.75 | 21.61 | 1.08 | 97.74 | 93.53 | 99.34 | 84.34 | 69.67 |
| *SmMYB113*-OE1-Pulp3 | 21.75 | 21.61 | 1.08 | 97.88 | 93.97 | 99.37 | 84.4 | 70.45 |
| *SmMYB113*-OE4-Peel1 | 21.75 | 21.67 | 1.08 | 97.85 | 93.87 | 99.63 | 85.21 | 72.07 |
| *SmMYB113*-OE4-Peel2 | 21.75 | 21.65 | 1.08 | 97.73 | 93.57 | 99.57 | 85.02 | 71.81 |
| *SmMYB113*-OE4-Peel3 | 21.75 | 21.65 | 1.08 | 97.66 | 93.42 | 99.54 | 84.57 | 70.91 |
| *SmMYB113*-OE4-Pulp1 | 21.75 | 21.61 | 1.08 | 97.62 | 93.27 | 99.38 | 83.7 | 70.9 |
| *SmMYB113*-OE4-Pulp2 | 21.75 | 21.6 | 1.08 | 97.74 | 93.6 | 99.34 | 83.76 | 70.22 |
| *SmMYB113*-OE4-Pulp3 | 21.75 | 21.6 | 1.08 | 97.82 | 93.81 | 99.32 | 83.79 | 70.91 |
| Average | 21.71 | 21.60 | 1.08 | 97.79 | 93.73 | 99.49 | 83.74 | 71.40 |

**Table S3 The information of 27 novel genes related to color difference and 32 novel genes involved in anthocyanin biosynthesis regulated by SmMYB113**

| Gene ID | The annotation in NCBI database |
| --- | --- |
| The 27 novel genes related to color difference | |
| SMEL_003g182990.1 | XP_006353906.1\|1.9e-272\|PREDICTED: protein DETOXIFICATION 35-like [Solanum tuberosum] |
| SMEL_004g218850.1 | XP_006362070.1\|3.5e-70\|PREDICTED: cytochrome b5-like [Solanum tuberosum] |
| SMEL_007g292660.1 | NP_001274785.1\|1.6e-130\|pectin methyl esterase [Solanum tuberosum] |
| SMEL_000g059970.1 | XP_006348185.1\|5.2e-93\|PREDICTED: glutathione S-transferase F11 [Solanum tuberosum] |
| SMEL_009g331690.1 | NP_001289828.1\|2.1e-89\|flavonoid 3',5'-methyltransferase [Solanum lycopersicum] |
| SMEL_010g337320.1 | XP_006344141.1\|8.8e-267\|PREDICTED: 3-ketoacyl-CoA synthase 6-like [Solanum tuberosum] |
| SMEL_000g067480.1 | XP_019259257.1\|1.3e-278\|PREDICTED: phosphoethanolamine N-methyltransferase-like [Nicotiana attenuata] |
| SMEL_001g132410.1 | XP_016545143.1\|1.6e-94\|PREDICTED: uclacyanin-3-like [Capsicum annuum] |
| SMEL_000g054110.1 | XP_015061505.1\|6.0e-131\|aquaporin TIP2-1-like [Solanum pennellii] |
| SMEL_005g226620.1 | XP_004239963.1\|1.4e-119\|amino acid transporter AVT6C [Solanum lycopersicum] |
| SMEL_006g267910.1 | NP_001234761.2\|2.4e-146\|expansin18 precursor [Solanum lycopersicum] |
| SMEL_003g186140.1 | XP_015069712.1\|0.0e+00\|DNA-binding protein SMUBP-2 [Solanum pennellii] |
| SMEL_005g224290.1 | XP_006345582.2\|2.0e-221\|PREDICTED: probable polygalacturonase isoform X1 [Solanum tuberosum] |
| SMEL_010g357250.1 | P37122.1\|2.4e-296\|RecName: Full=Cytochrome P450 76A2; AltName: Full=CYPLXXVIA2; AltName: Full=Cytochrome P-450EG7 |
| SMEL_011g366680.1 | XP_015057078.1\|2.3e-221\|serine/threonine-protein kinase D6PKL2-like [Solanum pennellii] |
| SMEL_003g195180.1 | XP_006341483.1\|1.3e-204\|PREDICTED: transcription factor ICE1-like [Solanum tuberosum] |
| SMEL_005g224010.1 | XP_004240145.1\|8.3e-215\|glucose-6-phosphate/phosphate translocator 2, chloroplastic [Solanum lycopersicum] |
| SMEL_008g298320.1 | XP_006362485.1\|1.8e-146\|PREDICTED: BOI-related E3 ubiquitin-protein ligase 1-like [Solanum tuberosum] |
| SMEL_009g334170.1 | XP_006366846.1\|2.1e-110\|PREDICTED: myb family transcription factor APL-like [Solanum tuberosum] |
| SMEL_012g399270.1 | XP_006340130.1\|2.9e-168\|PREDICTED: uncharacterized protein LOC102597591 [Solanum tuberosum] |
| SMEL_007g291050.1 | XP_004243411.1\|4.6e-157\|bifunctional riboflavin kinase/FMN phosphatase-like [Solanum lycopersicum] |
| SMEL_003g170420.1 | XP_006343233.2\|3.0e-31\|PREDICTED: uncharacterized protein LOC102599015 [Solanum tuberosum] |
| SMEL_010g358070.1 | XP_022842436.1\|5.9e-238\|exocyst complex component EXO70A1-like [Olea europaea var. sylvestris] |
| SMEL_002g162860.1 | XP_006357188.1\|0.0e+00\|PREDICTED: probable inactive leucine-rich repeat receptor-like protein kinase At3g03770 [Solanum tuberosum] |
| SMEL_012g396370.1 | XP_016440571.1\|3.3e-35\|PREDICTED: uncharacterized protein LOC107766324 [Nicotiana tabacum] |
| SMEL_008g316440.1 | XP_015084575.1\|1.4e-74\|ethylene-responsive transcription factor ERF038-like [Solanum pennellii] |
| SMEL_012g396960.1 | XP_016576957.1\|2.9e-157\|PREDICTED: acylsugar acyltransferase 3-like [Capsicum annuum] |
| The 32 novel genes involved in anthocyanin biosynthesis regulated by SmMYB113 | |
| SMEL_000g011930.1 | XP_006357941.1\|3.0e-83\|PREDICTED: ribonuclease 3-like protein 3 [Solanum tuberosum] |
| SMEL_000g027530.1 | XP_006339865.1\|1.9e-174\|PREDICTED: protein IRX15-LIKE-like [Solanum tuberosum] |
| SMEL_000g032180.1 | XP_006340805.1\|7.3e-250\|PREDICTED: uncharacterized protein LOC102584459 isoform X1 [Solanum tuberosum] |
| SMEL_000g059970.1 | XP_006348185.1\|5.2e-93\|PREDICTED: glutathione S-transferase F11 [Solanum tuberosum] |
| SMEL_000g063890.1 | XP_015067245.1\|0.0e+00\|external alternative NAD(P)H-ubiquinone oxidoreductase B2, mitochondrial [Solanum pennellii] |
| SMEL_000g092740.1 | XP_015067064.1\|9.6e-285\|cytochrome P450 710A11 [Solanum pennellii] |
| SMEL_000g100040.1 | XP_006365227.1\|3.7e-262\|PREDICTED: polygalacturonase-1 non-catalytic subunit beta [Solanum tuberosum] |
| SMEL_001g117930.1 | XP_016539699.1\|2.7e-105\|PREDICTED: cold-regulated 413 plasma membrane protein 2 isoform X1 [Capsicum annuum] |
| SMEL_001g117970.1 | XP_006346509.1\|0.0e+00\|PREDICTED: extra-large guanine nucleotide-binding protein 1-like [Solanum tuberosum] |
| SMEL_001g127150.1 | XP_006347771.1\|3.6e-156\|PREDICTED: annexin D4-like isoform X1 [Solanum tuberosum] |
| SMEL_001g133130.1 | XP_006354629.1\|2.0e-165\|PREDICTED: uncharacterized protein LOC102583227 [Solanum tuberosum] |
| SMEL_001g138020.1 | XP_004229656.1\|1.7e-91\|uncharacterized protein LOC101254731 [Solanum lycopersicum] |
| SMEL_001g153070.1 | XP_004228722.1\|1.7e-91\|protein YLS3 [Solanum lycopersicum] |
| SMEL_003g182990.1 | XP_006353906.1\|1.9e-272\|PREDICTED: protein DETOXIFICATION 35-like [Solanum tuberosum] |
| SMEL_003g193380.1 | XP_006364189.1\|0.0e+00\|PREDICTED: uncharacterized protein LOC102590844 isoform X1 [Solanum tuberosum] |
| SMEL_004g206760.1 | XP_006351875.1\|2.4e-254\|PREDICTED: nodulation-signaling pathway 2 protein [Solanum tuberosum] |
| SMEL_004g218850.1 | XP_006362070.1\|3.5e-70\|PREDICTED: cytochrome b5-like [Solanum tuberosum] |
| SMEL_004g219460.1 | PHT30476.1\|1.4e-181\|Heat stress transcription factor B-4 [Capsicum baccatum] |
| SMEL_005g234690.1 | XP_006367560.1\|7.2e-242\|PREDICTED: anthocyanidin 3-O-glucosyltransferase [Solanum tuberosum] |
| SMEL_005g236240.1 | XP_006367986.2\|3.8e-240\|PREDICTED: acetyl-CoA-benzylalcohol acetyltransferase-like [Solanum tuberosum] |
| SMEL_005g236910.1 | XP_019066841.1\|8.8e-105\|BRI1 kinase inhibitor 1 [Solanum lycopersicum] |
| SMEL_005g238370.1 | XP_006349903.1\|1.1e-261\|PREDICTED: crocetin glucosyltransferase, chloroplastic-like [Solanum tuberosum] |
| SMEL_005g239710.1 | XP_006342466.1\|1.1e-140\|PREDICTED: probable WRKY transcription factor 17 [Solanum tuberosum] |
| SMEL_005g241500.1 | XP_006349881.1\|7.2e-185\|PREDICTED: chitinase-like protein 1 [Solanum tuberosum] |
| SMEL_006g251030.1 | NP_001333898.1\|3.2e-177\|protein phosphatase 2C [Solanum lycopersicum] |
| SMEL_006g251500.1 | XP_006363330.1\|2.0e-274\|PREDICTED: protein DETOXIFICATION 35-like [Solanum tuberosum] |
| SMEL_007g285730.1 | XP_015081173.1\|5.3e-170\|F-box protein CPR1-like [Solanum pennellii] |
| SMEL_009g321740.1 | NP_001275094.1\|9.9e-141\|single-stranded DNA binding protein precursor [Solanum tuberosum] |
| SMEL_009g331690.1 | NP_001289828.1\|2.1e-89\|flavonoid 3',5'-methyltransferase [Solanum lycopersicum] |
| SMEL_010g339330.1 | XP_006352038.2\|8.1e-168\|PREDICTED: peroxidase 27-like [Solanum tuberosum] |
| SMEL_011g374100.1 | XP_015167636.1\|6.5e-243\|PREDICTED: zeatin O-xylosyltransferase-like [Solanum tuberosum] |
| SMEL_012g396960.1 | XP_016576957.1\|2.9e-157\|PREDICTED: acylsugar acyltransferase 3-like [Capsicum annuum] |
