# Supplementary material for: RNA-sequencing analysis reveals novel genes involved in the different peel color formation in eggplant
Source: Hortic Res. 2023 Sep 4;10(10):uhad181. doi: 10.1093/hr/uhad181 (PMC10599318; doi:10.1093/hr/uhad181)
Supplement: Web_Material_uhad181 [file web_material_uhad181.zip › Supplementary Figures.docx]

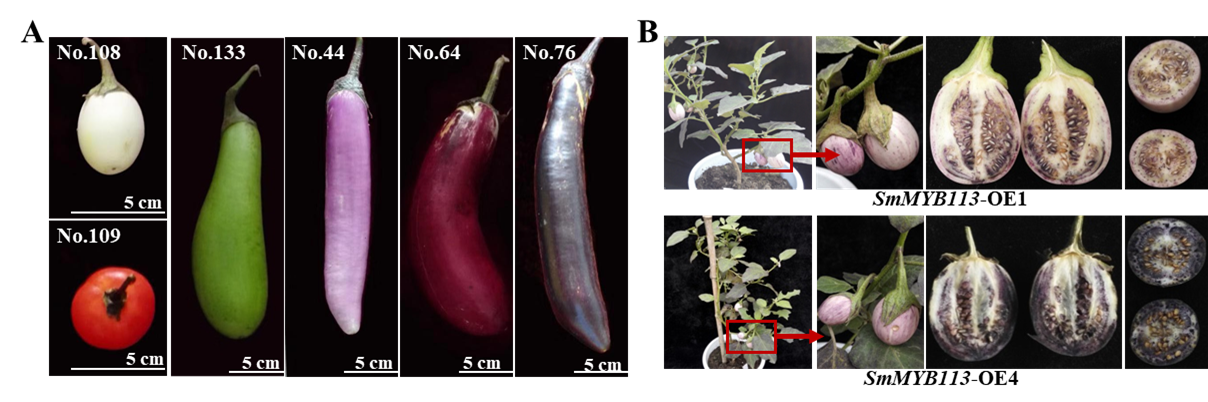


**Fig. S1 The phenotypes of the six eggplant cultivars and the *SmMYB113-*overexpressing transgenic eggplant lines.**


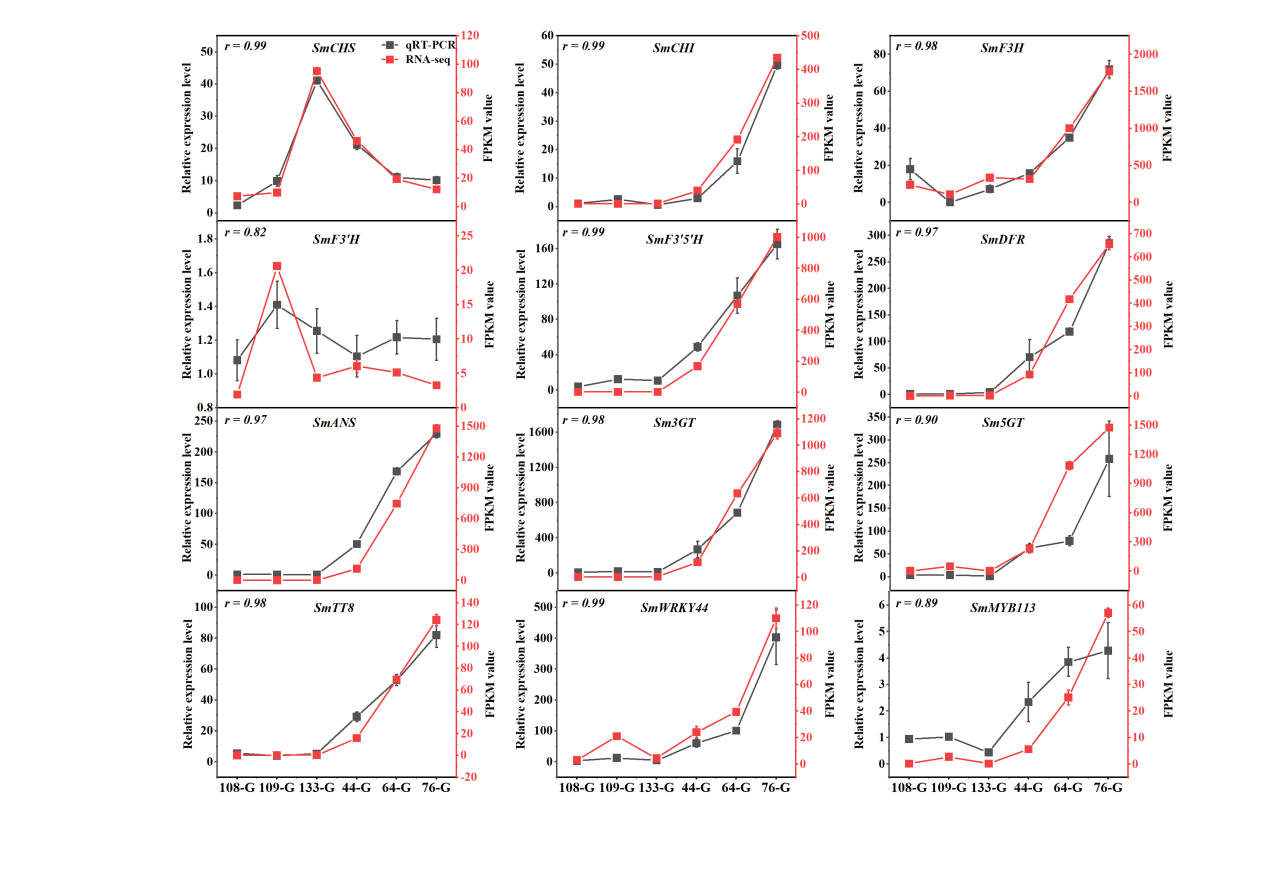


**Fig. S2 Correlation analysis of the RNA-seq data and qRT-PCR data.**


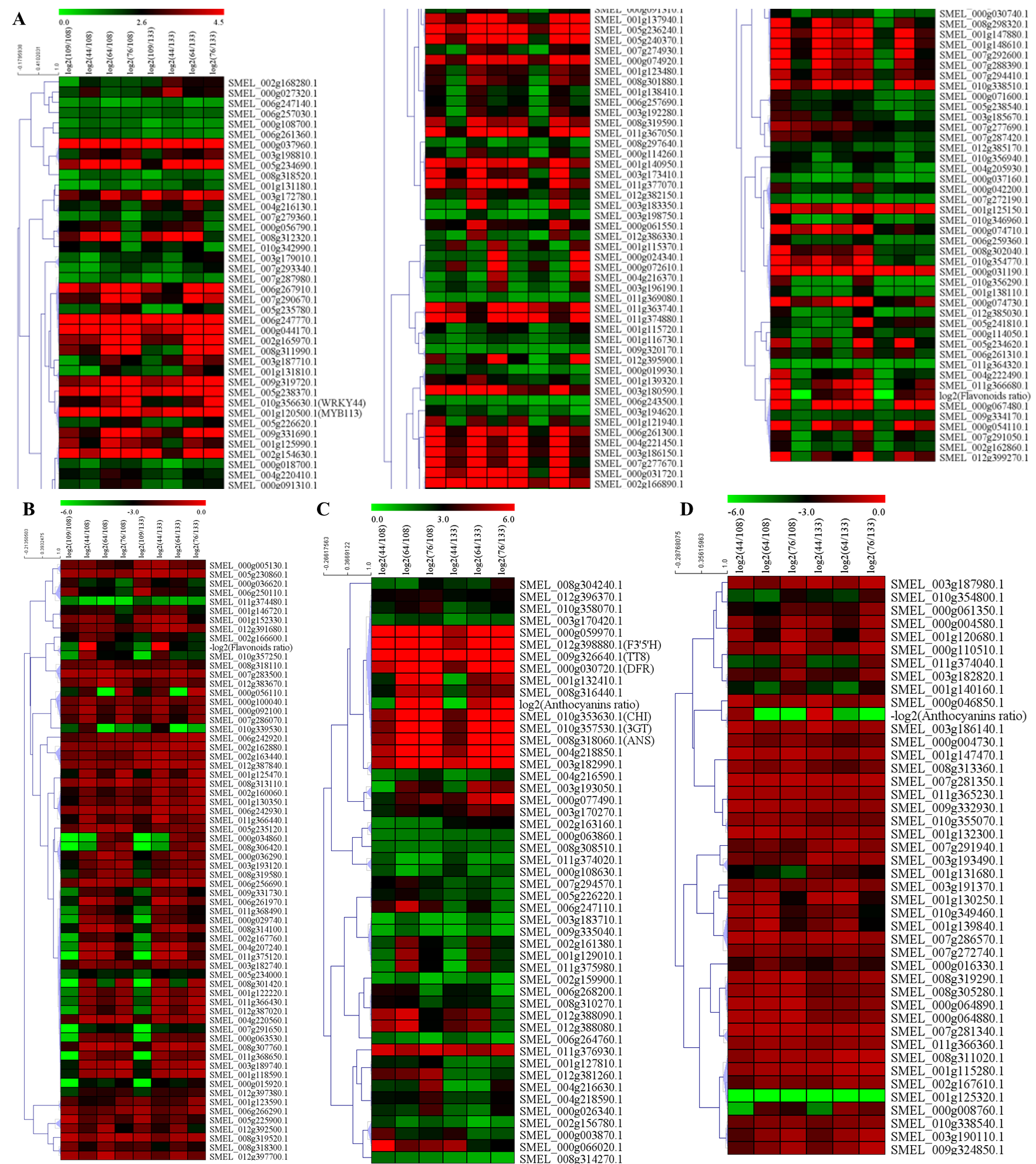


**Fig. S3 HCL analysis of DEGs with the content of flavonoids and anthocyanins based on the RNA-seq data.** The correlation of the 129 commonly up-regulated (A) and 65 commonly down-regulated (B) DEGs correlated with flavonoid biosynthesis. The correlation of the 48 commonly up-regulated (C) and 43 commonly down-regulated (D) DEGs with anthocyanin biosynthesis.


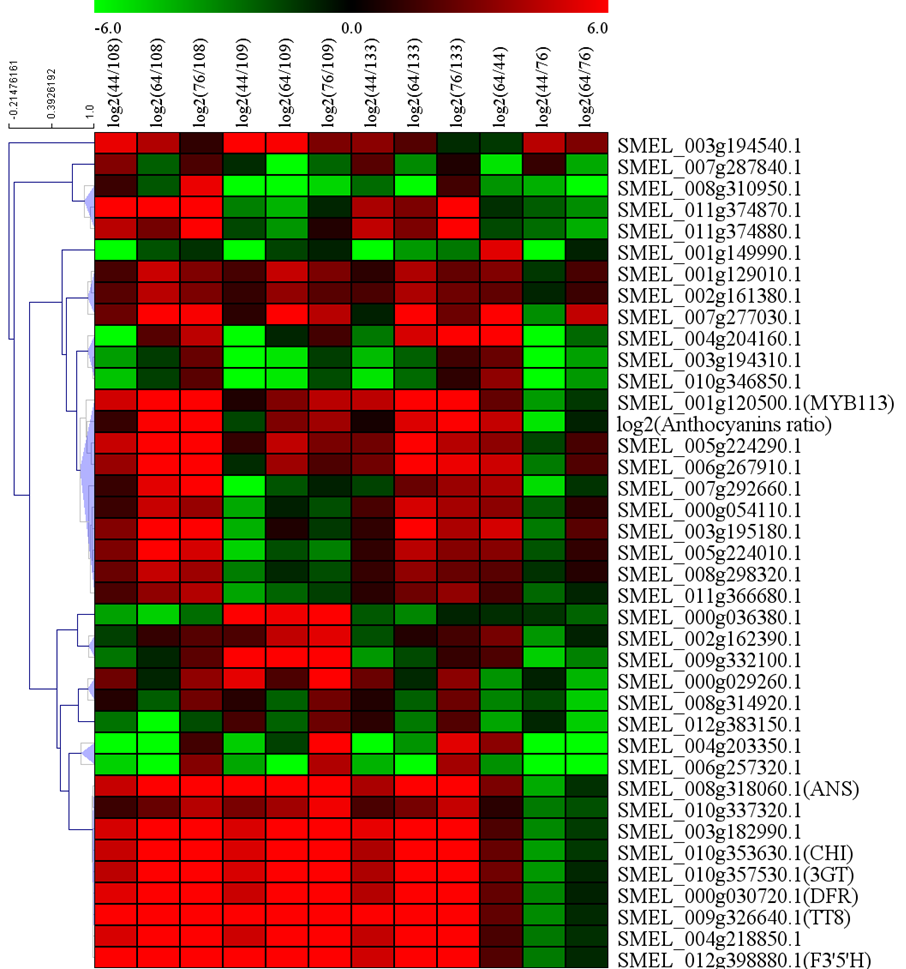


**Fig. S4 HCL analysis of DEGs related to the anthocyanin content and anthocyanin biosynthesis-related genes based on the RNA-seq data.**


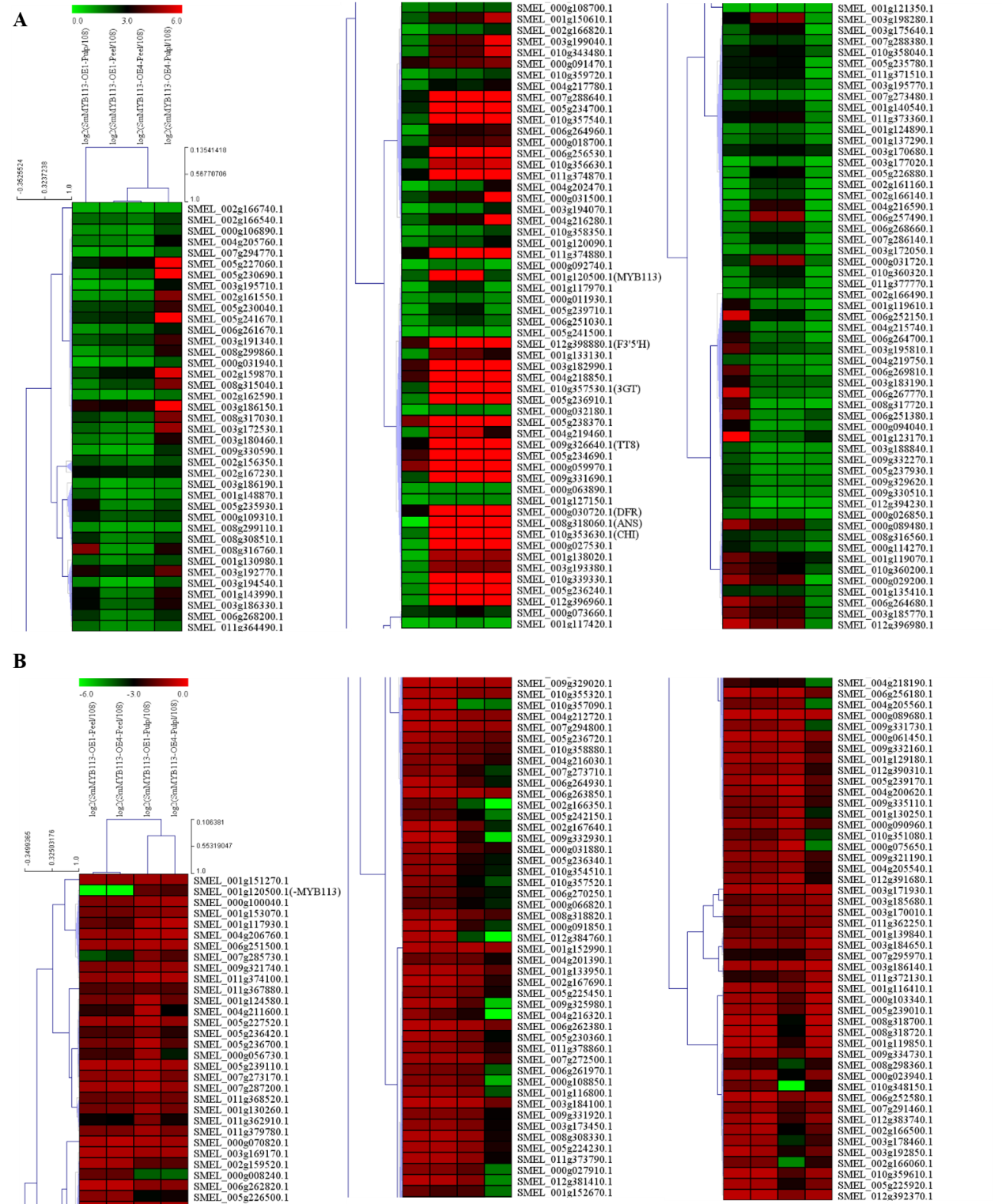


**Fig. S5 Hierarchical clustering analysis of up-regulated (A) and down-regulated (B) DEGs, as well as anthocyanin biosynthesis-related genes based on the RNA-seq data.**


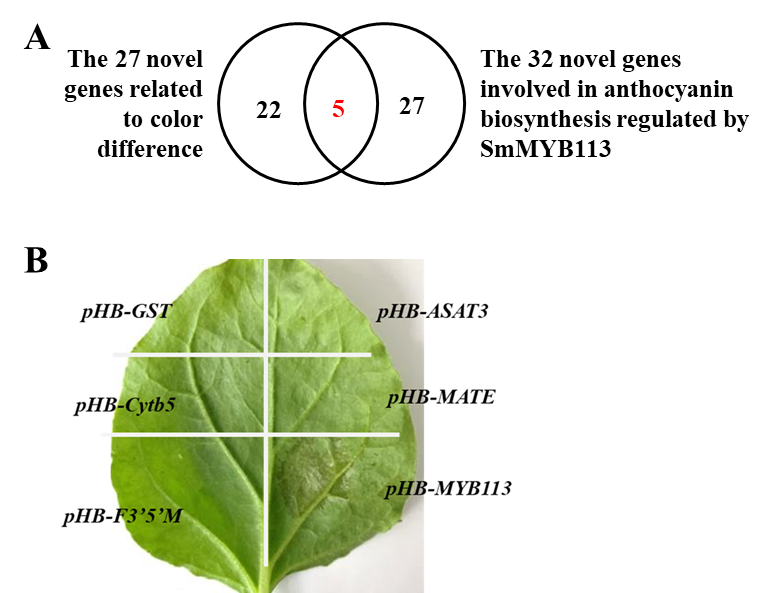


**Fig. S6** **Venn analysis (A) and functional characterization of the roles of the five novel genes in tobacco leaf using transient expression assay (B).**
